# Prediction of delayed graft function after kidney transplantation: comparison between logistic regression and machine learning methods

**DOI:** 10.1186/s12911-015-0206-y

**Published:** 2015-10-14

**Authors:** Alexander Decruyenaere, Philippe Decruyenaere, Patrick Peeters, Frank Vermassen, Tom Dhaene, Ivo Couckuyt

**Affiliations:** Department of Nephrology, Ghent University Hospital, Ghent, Belgium; Department of Thoracic and Vascular Surgery, Ghent University Hospital, Ghent, Belgium; Department of Information Technology (INTEC), Ghent University - iMinds, Ghent, Belgium

**Keywords:** Decision trees, Delayed graft function, Discriminant analysis, Kidney transplantation, Logistic models, Machine learning, Predictive analysis, ROC curve, Sensitivity and specificity, Support vector machines

## Abstract

**Background:**

Predictive models for delayed graft function (DGF) after kidney transplantation are usually developed using logistic regression. We want to evaluate the value of machine learning methods in the prediction of DGF.

**Methods:**

497 kidney transplantations from deceased donors at the Ghent University Hospital between 2005 and 2011 are included. A feature elimination procedure is applied to determine the optimal number of features, resulting in 20 selected parameters (24 parameters after conversion to indicator parameters) out of 55 retrospectively collected parameters. Subsequently, 9 distinct types of predictive models are fitted using the reduced data set: logistic regression (LR), linear discriminant analysis (LDA), quadratic discriminant analysis (QDA), support vector machines (SVMs; using linear, radial basis function and polynomial kernels), decision tree (DT), random forest (RF), and stochastic gradient boosting (SGB). Performance of the models is assessed by computing sensitivity, positive predictive values and area under the receiver operating characteristic curve (AUROC) after 10-fold stratified cross-validation. AUROCs of the models are pairwise compared using Wilcoxon signed-rank test.

**Results:**

The observed incidence of DGF is 12.5 %. DT is not able to discriminate between recipients with and without DGF (AUROC of 52.5 %) and is inferior to the other methods. SGB, RF and polynomial SVM are mainly able to identify recipients without DGF (AUROC of 77.2, 73.9 and 79.8 %, respectively) and only outperform DT. LDA, QDA, radial SVM and LR also have the ability to identify recipients with DGF, resulting in higher discriminative capacity (AUROC of 82.2, 79.6, 83.3 and 81.7 %, respectively), which outperforms DT and RF. Linear SVM has the highest discriminative capacity (AUROC of 84.3 %), outperforming each method, except for radial SVM, polynomial SVM and LDA. However, it is the only method superior to LR.

**Conclusions:**

The discriminative capacities of LDA, linear SVM, radial SVM and LR are the only ones above 80 %. None of the pairwise AUROC comparisons between these models is statistically significant, except linear SVM outperforming LR. Additionally, the sensitivity of linear SVM to identify recipients with DGF is amongst the three highest of all models. Due to both reasons, the authors believe that linear SVM is most appropriate to predict DGF.

## Background

Kidney transplantation is the preferred treatment for patients with end-stage renal disease, improving survival, cardiovascular comorbidity and quality of life [1, 2]. Unfortunately, not every transplanted kidney is functioning properly at the beginning. When ischemia/reperfusion injury is the cause of this early postoperative graft dysfunction, the term ‘delayed graft function’ (DGF) is used [3, 4]. DGF is diagnosed clinically after exclusion of other possible causes of early graft dysfunction, such as vascular thrombosis or hyperacute rejection [4, 5]. It is usually defined as the need for dialysis within the first week after transplantation [4].

Despite advances in pretreatment of donors and recipients, as well as in diagnostic and therapeutic modalities, the incidence of DGF has not decreased, nor have its short-term and long-term effects [6]. The incidence is possibly increasing, which might partly be explained by using more expanded-criteria donors and donors after cardiac death, as well as by selecting more recipients who are possibly more prone to DGF. The incidence of DGF with deceased donors varies from 2 to 50 %, depending on country, transplant center and the definition used. The incidence of DGF with living donors is lower and varies from 4 to 10 % [7].

The short-term and long-term consequences of DGF are increasingly being documented. Firstly, DGF has an adverse impact on the immediate post-transplant course by causing prolonged hospitalization and rehabilitation, and higher transplantation costs [8, 9]. Secondly, it is associated with an increased rate of acute rejection and with reduced long-term graft function [10]. Finally, it leads to long-term graft loss [10], independent of the increased risk of acute rejection [11, 12], and reduced patient survival [13].

Because of the deleterious consequences, several predictive models for DGF have been developed within the last few years. To date, four risk prediction models have been developed using logistic regression [14–17]. However, machine learning methods are also effective to detect new risk factors and to achieve acceptable predictive accuracy [18, 19]. Brier et al. [20] and Santori et al. [21] have already demonstrated that neural networks have higher sensitivity but lower specificity than logistic regression in the prediction of DGF. Other studies suggest that neural networks [22] and tree-based models [23] also have higher sensitivity but lower specificity than Cox regression in the prediction of graft survival. Consistently, another tree-based model [24] and a Bayesian belief network [25] achieve reasonable predictive accuracy for graft survival.

In this study, the goal is therefore to analyze and discuss the performance of different modeling techniques in the prediction of DGF and to identify which method is most suited to the task at hand.

## Methods

### Study cohort

The study cohort consists of consecutive adults (≥18 years) undergoing kidney transplantation from deceased donors at the Ghent University Hospital between January 1^st^, 2005 and December 31^st^, 2011. A total of 508 transplantations are performed. After exclusion of 11 transplantations, the study cohort consists of 497 transplantations. Reasons for exclusion are death of recipient or graft loss within the first week after transplantation. DGF is defined as the need for dialysis within the first week after transplantation. This study is conducted in accordance with the Declaration of Helsinki and is approved by the Ethics Committee of Ghent University Hospital. Due to the retrospective nature of this study, the need for informed consent is waived.

Fifty-five parameters are retrospectively collected as potential risk factors for DGF. Parameters related to donor include age, sex, body mass index, cytomegalovirus serology, length of stay in intensive care unit, terminal serum creatinine, subtype, terminal urine output, terminal systolic and diastolic blood pressure, pretreatment with dopamine/dobutamine/epinephrine/norepinephrine, terminal central venous pressure, diabetes mellitus, history of hypertension, hypotensive episodes during pre-explantation period, graft atherosclerosis (assessment by explant surgeon), and graft quality (assessment by explant surgeon). Parameters related to preservation and operation include preservation method, preservation solution, cold ischemia time, warm ischemia time, perioperative diuresis, perioperative graft reperfusion, donor-recipient sex, and donor-recipient cytomegalovirus serology. Parameters related to recipient include age, sex, ethnicity, body mass index, cytomegalovirus serology, modality and duration of dialysis, panel reactive antibodies at time of transplantation and peak panel reactive antibodies, number of previous kidney transplantations, human leukocyte antigen mismatches, preoperative systolic and diastolic blood pressure at time of transplantation, diabetes mellitus, lipid levels (triglycerides, total cholesterol, high-density lipoprotein and low-density lipoprotein) at time of transplantation, pulmonary hypertension (systolic pulmonary artery pressure >35 mmHg during pretransplant evaluation period), iliac artery atheromatosis or stenosis (imaging studies during pretransplant evaluation period or assessment during transplantation), reduced cardiac function (ejection fraction <40 % during pretransplant evaluation period using echocardiography or coronary catheterization), impaired effective circulating volume (clinical assessment at time of transplantation), abdominal compartment syndrome (clinical assessment at time of transplantation), anti-thymocyte globulin induction therapy, acute calcineurin inhibitor toxicity (serum level above the recommended therapeutic range), urinary tract obstruction (assessment by surgeon during revision), and pretransplant transfusion.

Categorical parameters with more than two possible values are converted to indicator parameters (dummy variables) as required by most of the predictive models.

### Feature selection

Feature (or variable) selection is a process of determining a subset of relevant parameters with respect to the predictive models. Many parameters might be irrelevant or contribute very little to the predictive models. Irrelevant parameters can actually degrade the prediction. Hence, it is crucial to make a good selection of the most influential subset of parameters.

In this study a recursive feature elimination procedure is used based on 10-fold stratified cross-validation [26]. The relative importance of the features is ranked using an external model, i.e., the coefficients of a logistic regression model. The full feature set is then iteratively pruned by removing the feature with the lowest importance until the 10-fold stratified cross-validation score decreases significantly, resulting in 24 selected parameters (two categorical parameters out of 20 selected parameters both have three possible values and are converted to three indicator parameters, resulting in a total of 24 selected parameters).

### Statistical models

The reduced data set of 24 parameters is fitted using 9 distinct types of predictive models: logistic regression, linear discriminant analysis, quadratic discriminant analysis, support vector machines (using linear, radial basis function and polynomial kernels), decision tree, random forest and stochastic gradient boosting. An exhaustive grid search is used based on 10-fold stratified cross-validation to determine the optimal hyper-parameters of each predictive model. The hyper-parameters that are optimized are presented in Table 1 with the optimal values in bold. The hyper-parameters that are not described in this table are set to the default values used in the scikit-learn library [27].

**Table 1:** Optimal hyper-parameters after exhaustive grid search

Statistical method	Hyper-parameter	Values
Decision tree	Class weights	auto, 0 to 0.20 and 1 to 0.80, 0 to 0.10 and 1 to 0.90, 0 to 0.05 and 1 to 0.95
	Maximum depth	1 to 10 (8)
	Minimum samples split	2 to nVars+1 (18)
	Maximum features	auto, sqrt, log2
Random forest	Number of estimators	1000
	Class weights	auto, 0 to 0.20 and 1 to 0.80, 0 to 0.10 and 1 to 0.90, 0 to 0.05 and 1 to 0.95
	Maximum depth	1 to 10 (9)
	Minimum samples split	2 to nVars+1 (24)
	Maximum features	auto, sqrt, log2
Random forest (full)	Number of estimators	1000
	Class weights	auto, 0 to 0.20 and 1 to 0.80, 0 to 0.10 and 1 to 0.90, 0 to 0.05 and 1 to 0.95
	Maximum depth	1 to 10 (1)
	Minimum samples split	2 to nVars+1 (63)
	Maximum features	auto, sqrt, log2
Gradient boosting	Number of estimators	1000
	Maximum depth	1 to 10 (1)
	Minimum samples split	2 to nVars+1 (9)
	Maximum features	auto, sqrt, log2
	Learning rate	0.1, 0.05, 0.02, 0.01
LDA	Number of components	None or 1 to nVars +1
QDA	Regularizing parameter	0 to 1 (0.89)
Linear SVM	Class weights	auto, 0 to 0.20 and 1 to 0.80, 0 to 0.10 and 1 to 0.90, 0 to 0.05 and 1 to 0.95
	C	0.001, 0.01, 0.1, 1, 10, 100, 1000
Radial SVM	Class weights	auto, 0 to 0.20 and 1 to 0.80, 0 to 0.10 and 1 to 0.90, 0 to 0.05 and 1 to 0.95
	C	0.001, 0.01, 0.1,1, 10, 100, 1000
	Gamma	0.1, 0.01, 0.001, 0.0001
Polynomial SVM	Class weights	auto, 0 to 0.20 and 1 to 0.80, 0 to 0.10 and 1 to 0.90, 0 to 0.05 and 1 to 0.95
	C	0.001, 0.01, 0.1,1, 10, 100, 1000
	Gamma	0.1, 0.01, 0.001, 0.0001
Logistic regression	Class weights	auto, 0 to 0.20 and 1 to 0.80, 0 to 0.10 and 1 to 0.90, 0 to 0.05 and 1 to 0.95
	C	0.001, 0.01, 0.1,1, 10, 100, 1000

The hyper-parameters that are not described in this table are set to the default values used in the scikit-learn library [27]

*Abbreviations*: *LDA* linear discriminant analysis, *QDA* quadratic discriminant analysis, *SVM* support vector machine

Logistic regression (LR) is a linear model that assumes that the targets follow a Gaussian distribution. A prediction on a transplantation x is made using y(x) = *w*^T^x, where *w* is the weight vector being learned.

Linear discriminant analysis (LDA) produces an optimally weighted linear function of chosen log-transformed markers and the discriminating threshold value minimizes the expected number of misclassifications under the normal model.

Quadratic discriminant analysis (QDA) is related to LDA. Unlike LDA however, there is no assumption that the covariance of each class is identical. This produces a quadratic discriminant function, which contains second order terms.

Support vector machines (SVMs) are sparse kernel machines, a type of models that rely only on a subset of the data (the support vectors) to predict unknown class labels. SVMs separate input data using a good-fitting hyperplane. Kernels can be used to transform this hyperplane into a non-linear input separator. We chose a linear, a radial basis function and a polynomial kernel.

A decision tree (DT) separates the data (the parent node) into two subsets (the child nodes) by the best splitting feature. The two resulting subsets become the new parent nodes, which are subsequently split further into two child nodes. This procedure continues until all observations are classified.

Random forest (RF) is an ensemble machine learning method based on the construction of multiple decision trees. The main underlying technique is bootstrap aggregating (bagging). In each decision tree, a data point falls into a particular leaf depending on its features and is assigned a prediction. The predictions of the data points are then averaged. RF has a built-in feature selection system and allows for joint features, making it not only an additive model but also a multiplicative one.

Stochastic gradient boosting (SGB) constructs additive regression tree models sequentially to fit pseudo-residuals of previous cumulative models. This stepwise manner combines the performance of weak learners (i.e., regression trees here) iteratively into a strong learner with high accuracy.

As RF has a built-in feature selection system, the full data set of all collected parameters is also fitted using RF. By doing this, we can compare the performance between the RF fitted on the reduced data set and the RF fitted on the full data set, to evaluate if the recursive feature elimination procedure influences the built-in feature selection of RF.

### Model validation

Performance of the models is assessed by computing the diagnostic test characteristics, including sensitivity and positive predictive value (PPV), and by evaluating the discriminative capacity, using the area under the receiver operating characteristic curve (AUROC), which measures how well the relative ranking of the individual risk is in substantially the correct order (observed incidence in those with higher predicted risks are higher).

10-fold stratified cross-validation is used to obtain a better generalization estimate of the performance. In 10-fold stratified cross-validation, the data set is partitioned into ten equal size folds such that each fold contains roughly the same proportion of ‘DGF’ and ‘no DGF’ class labels. Of the ten folds, a single fold is retained as the validation data for testing the model, and the remaining nine folds are used as training data. The cross-validation process is then repeated ten times, with each of the ten folds used exactly once as the validation data. The ten results from the folds are averaged to produce a single estimation. The advantage is that all observations are used for both training and validation, and each observation is used for validation exactly once.

### Model comparison

Subsequently, the models are pairwise compared. For each model, the AUROC is computed in each of the ten folds. The ten values for the AUROC of one model are compared with the values of another model using the two-sided Wilcoxon signed-rank test at 5 % significance level.

All computations are carried out using Python, specifically in the SciPy environment using the scikit-learn library [27]. Continuous data are presented as mean ± standard deviation and categorical data are reported as percentages. Counts are put in parentheses.

## Results

### Descriptive statistics

The most relevant donor, preservation/operation, and recipient characteristics are presented in Table 2. After exclusion, 497 transplantations are used for the analysis, consisting of 432 unique donors (362 donated a single kidney to a recipient of our center and the other kidney to a recipient of a different center, 5 donated both kidneys to the same recipient of our center, and 65 donated both kidneys to different recipients of our center) and 496 unique recipients (1 recipient underwent two kidney transplantations at different times from deceased donors during the study period). The observed incidence of DGF is 12.5 % (62/497).

**Table 2:** Baseline characteristics (*n* = 497)

Donor	
Sex	
male	60.4 % (300)
female	39.6 % (197)
Subtype	
DBD	90.3 % (449)
DCD	9.7 % (48)
Age (year)	42.6 ± 14.77
Terminal SCr (mg/dL)	0.878 ± 0.4757
Preservation/Operation	
Preservation solution	
HTK	31.0 % (154)
HTK + UW	0.2 % (1)
UW	68.6 % (341)
missing	0.2 % (1)
CIT (hour)	14.19 ± 4.328
WIT (min)	22.3 ± 7.09
Recipient	
Sex	
male	66.6 % (331)
female	33.4 % (166)
Modality of dialysis	
hemodialysis	71.2 % (354)
peritoneal dialysis	22.7 % (113)
pre-emptive	6.0 % (30)
HLA mismatches	
0	8.9 % (44)
1	7.8 % (39)
2	26.4 % (131)
3	40.8 % (203)
4	10.9 % (54)
5	4.0 % (20)
6	1.2 % (6)
Age (year)	52.8 ± 11.68
Duration of dialysis (year)	2.7 ± 1.68
PRA at time of Tx (%)	2.7 ± 11.44

*Abbreviations*: *CIT* cold ischemia time, *DBD* donor after brain death, *DCD* donor after cardiac/circulatory death, *HLA* human leukocyte antigen, *HTK* histidine-tryptophan-ketoglutarate, *PRA* panel reactive antibody, *SCr* serum creatinine, *Tx* transplantation, *UW* University of Wisconsin, *WIT* warm ischemia time

This imbalance in the data set is addressed by assigning more weight to the ‘DGF’ class during the learning phase of the predictive models. Only 11 (categorical) parameters out of the 55 retrospectively collected parameters are incomplete and contain missing values for a number of transplantations. The most frequent occurring value, which is the ‘normal’ category, is used to fill in these missing values. This is a safe assumption, because ‘abnormal’ values for risk factors are more likely to be emphasized and registered in the electronic medical records. However, ‘normal’ values are not always routinely registered in the electronic medical records and are retrospectively considered as missing values.

### Model performance and comparison

**Fig. 1:**
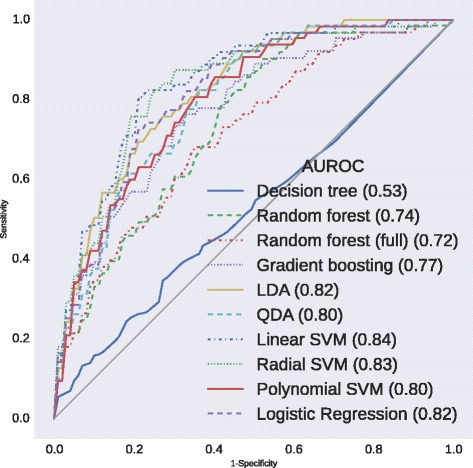
Receiver operating characteristic curves after 10-fold stratified cross-validation. *Abbreviations*: *AUROC* area under the receiver operating characteristic curve, *LDA* linear discriminant analysis, *QDA* quadratic discriminant analysis, *SVM* support vector machine

**Fig. 2:**
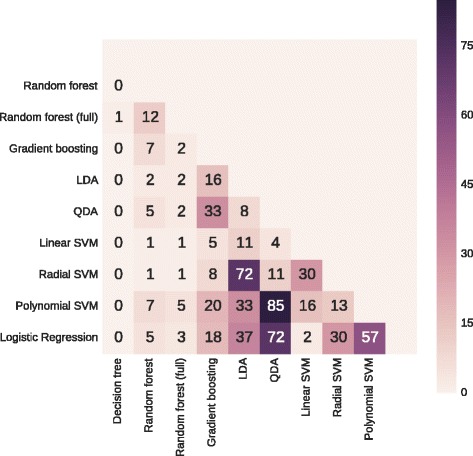
*P*-values (%) of pairwise model comparison using Wilcoxon signed-rank test. *Abbreviations*: *LDA* linear discriminant analysis, *QDA* quadratic discriminant analysis, *SVM* support vector machine

Diagnostic test characteristics and AUROCs after 10-fold stratified cross-validation are presented in Table 3. The receiver operating characteristic curves and the *p*-values of the pairwise AUROC comparisons are presented in Figs. 1 and 2, respectively. The selected features and their respective odds ratios (LR), Z-scores (linear SVM), and Gini index (RF fitted on the full data set) are presented in Table 4.

**Table 3:** Performance of the statistical methods after 10-fold stratified cross-validation

Statistical method	Sensitivity (%)		PPV (%)		AUROC (%)
	No DGF	DGF	No DGF	DGF	
Decision tree	75.4 ± 6.64	29.5 ± 16.29	88.2 ± 2.73	14.2 ± 8.13	52.5 ± 8.55
Gradient boosting	98.8 ± 1.55	16.2 ± 12.94	89.2 ± 1.67	58.3 ± 38.19	77.2 ± 9.64
Random forest	96.3 ± 4.05	16.4 ± 14.92	89.0 ± 2.09	43.9 ± 38.19	73.9 ± 9.94
Random forest (full)	100.0 ± 0.00	0.0 ± 0.00	87.5 ± 0.64	0.0 ± 0.00	71.6 ± 12.38
LDA	94.7 ± 2.92	27.6 ± 15.10	90.2 ± 2.00	42.3 ± 19.94	82.2 ± 6.14
QDA	89.9 ± 5.35	37.6 ± 17.26	91.0 ± 2.55	37.9 ± 20.82	79.6 ± 7.55
Linear SVM	72.0 ± 6.29	83.8 ± 7.51	96.9 ± 1.34	30.6 ± 5.60	84.3 ± 4.11
Radial SVM	57.9 ± 7.45	88.8 ± 7.38	97.2 ± 1.87	23.6 ± 4.14	83.3 ± 4.05
Polynomial SVM	97.5 ± 1.90	10.9 ± 12.20	88.5 ± 1.14	24.0 ± 24.17	79.8 ± 5.33
Logistic regression	65.0 ± 8.25	85.5 ± 8.94	96.9 ± 1.84	26.5 ± 4.75	81.7 ± 5.82

*Abbreviations*: *AUROC* area under the receiver operating characteristic curve, *DGF* delayed graft function, *LDA* linear discriminant analysis, *PPV* positive predictive value, *QDA* quadratic discriminant analysis, *SVM* support vector machine

**Table 4:** Weights of the selected features

Feature	Odds ratio (LR)^a^	Z-score (linear SVM)^a^	Gini index (RF)^b^	
Donor				
Age (per 1 year)	1.060	0.744	0.037	(#9)
BMI (per 1 kg/m^2^)	0.751	−1.700	0.023	(#20)
Terminal SCr (per 1 mg/dL)	6.512	1.126	0.024	(#17.5)
Hypotensive episodes: yes *vs.* no	1.784	0.165	0.001	(#48.5)
Diabetes mellitus: yes *vs.* no	0.013	−1.041	0.001	(#48.5)
History of hypertension: yes *vs.* no	3.585	0.940	0.011	(#28)
Donor after cardiac death: yes *vs.* no	25.789	1.534	0.080	(#1)
Preservation/Operation				
Machine perfusion: yes *vs.* no	0.003	−1.078	0.000	(#60)
Perioperative graft reperfusion^c^	0.740	−0.844	0.027	(#14.5)
Preservation solution				
HTK + UW	0.00005	−0.510	0.000	(#60)
UW	0.080	−1.557	0.016	(#25)
HTK	0.050	−1.725	0.007	(#32.5)
Male donor-to-female recipient: yes *vs.* no	0.352	−0.750	0.019	(#23)
Recipient				
BMI (per 1 kg/m^2^)	1.144	0.941	0.054	(#4)
Duration of dialysis (per 1 day)	1.0005	0.324	0.057	(#3)
PRA at time of Tx (per 1 %)	0.977	−0.557	0.008	(#30.5)
Peak PRA (per 1 %)	1.017	0.585	0.025	(#16)
Acute CNI toxicity: yes *vs.* no	22.044	0.964	0.007	(#32.5)
Reduced cardiac function: yes *vs.* no	5.570	0.897	0.033	(#13)
Impaired ECV: yes *vs.* no	0.003	−1.141	0.000	(#60)
Urinary tract obstruction: yes *vs.* no	6.638	0.942	0.004	(#38.5)
Iliac artery				
normal	1.520	0.221	0.001	(#48.5)
atheromatosis	2.389	0.573	0.006	(#34.5)
stenosis	28.465	0.948	0.037	(#9)

^a^Fitted on the reduced data set

^b^Fitted on the full data set. Tied rank amongst all 68 features is given in parentheses

^c^Perioperative graft reperfusion is an ordinal feature (poor – patchy – moderate – good)

*Abbreviations*: *BMI* body mass index, *CNI* calcineurin inhibitor toxicity, *ECV* effective circulating volume, *HTK* histidine-tryptophan-ketoglutarate, *LR* logistic regression, *PRA* panel reactive antibody, *RF* random forest, *SCr* serum creatinine, *SVM* support vector machine, *Tx* transplantation, *UW* University of Wisconsin

DT is not able to discriminate between recipients with and without DGF (AUROC of 52.5 %) and is inferior to the other methods.

As SGB and RF mainly have high sensitivity (98.8 and 96.3 %, respectively) and high PPVs (89.2 and 89.0 %, respectively) in identifying recipients without DGF, their discriminative capacity (AUROC of 77.2 and 73.9 %, respectively) is superior to DT. However, RF is still outperformed by LDA, QDA, linear SVM, radial SVM and LR. SGB is only outperformed by linear SVM.

LDA and QDA already have higher sensitivity in identifying recipients with DGF (27.6 and 37.6 %, respectively) and only slightly lower sensitivity in identifying recipients without DGF (94.7 and 89.9 %, respectively), resulting in higher discriminative capacity (AUROC of 82.2 and 79.6 %, respectively). Both LDA and QDA outperform DT and RF, but only QDA is inferior to linear SVM.

Amongst all methods used, linear SVM, radial SVM and LR have the highest sensitivity in identifying recipients with DGF (83.8, 88.8 and 85.5 %, respectively), at the expense of identifying recipients without DGF (72.0, 57.9 and 65.0 %, respectively). However, their capability to identify both outcomes is reflected in a strong discriminative capacity (AUROC of 84.3, 83.3 and 81.7 %, respectively). Linear SVM outperforms each method, except for radial SVM, polynomial SVM and LDA. Radial SVM and LR outperform DT and RF, but only LR is inferior to linear SVM.

The performance of polynomial SVM is similar to that of SGB and RF, with high sensitivity (97.5 %) and high PPV (88.5 %) in identifying recipients without DGF, resulting in an AUROC of 79.8 %. Polynomial SVM also outperforms DT. Unlike SGB and RF however, it is not inferior to any of the methods used.

RF fitted on the full data set has a sensitivity of 100 % and a PPV of 87.5 % in identifying recipients without DGF, resulting in an AUROC of 71.6 %. It is superior to DT, which is fitted on the reduced data set and has no discriminative capacity, and non-inferior to RF fitted on the reduced data set. However, RF fitted on the full data set is inferior to each of the other methods used.

## Discussion

The risk prediction of DGF may be important in preventing its deleterious short-term and long-term consequences. To date, four predictive models are developed as a clinical tool to quantify the risk for DGF [14–17]. All models are developed using LR. We compared in this study several machine learning methods, including LR, in terms of their predictive accuracy for DGF. There are no studies that have used DT, SGB, RF, LDA, QDA or SVM in the prediction of DGF.

In our study, DT is not able to discriminate between recipients with and without DGF, and is inferior to the other methods. SGB, RF and polynomial SVM are mainly able to identify recipients without DGF and only outperform DT. Despite lower sensitivity in varying degrees to identify recipients without DGF, LDA, QDA, radial SVM and LR also have the ability to identify recipients with DGF, resulting in higher discriminative capacity, which outperforms DT and RF. Linear SVM has the highest discriminative capacity (AUROC of 84.3 %), outperforming each method, except for radial SVM, polynomial SVM and LDA. However, it is the only method superior to LR.

The AUROC focuses solely on the predictive accuracy of a model. As such, it cannot tell us whether the model is worth using in clinical practice, because it does not incorporate information on consequences. The method with maximal accuracy is not necessarily the best to choose. This choice should depend on the disadvantages or costs of not identifying a recipient with DGF as opposed to incorrectly predicting DGF in a recipient who will not develop it [28]. The advantages of an early hypothetic treatment should be weighed against possible iatrogenic damage and unnecessary additional costs. If we assume that the damage of an unnecessary treatment of DGF (a false-positive result) is limited, a more sensitive method should be used. If an unnecessary treatment is harmful, a more specific method should be used. Of course the trade-off between sensitivity and specificity should be kept in mind: a very sensitive method is useless when it is not specific enough and vice versa [29].

Currently, the management of DGF consists of a careful follow-up. Besides sonographic evaluation and precise biochemical monitoring, a biopsy is often performed, which is costly and invasive, possibly damaging the graft. Because of the complex and multifactorial characteristics of DGF, a standard therapy or drug does not yet exist [30]. Although a biopsy might be harmful, this is outweighed by the potential benefit of an early management, because DGF has deleterious short-term and long-term consequences. To date, a more sensitive method is therefore preferred. In our study, linear SVM, radial SVM and LR have the highest sensitivity in identifying recipients with DGF (83.8, 88.8 and 85.5 %, respectively).

To sum up, the discriminative capacities of LDA, linear SVM, radial SVM and LR are the only ones above 80 % (82.2, 84.3, 83.3 and 81.7 %, respectively). None of the pairwise AUROC comparisons between these models is statistically significant, except linear SVM outperforming LR. Additionally, a method with higher sensitivity is preferred over a method with higher specificity in the prediction of DGF. The sensitivity of linear SVM to identify recipients with DGF (83.8 %) is amongst the three highest of all methods used. Only radial SVM and LR have a slightly higher sensitivity (88.8 and 85.5 %, respectively). Due to both reasons, the authors believe that linear SVM is most appropriate to predict DGF.

72.0 % of the recipients who will not develop DGF are identified. These recipients can undergo the kidney transplantation without the need for a more precise monitoring. Only 3.1 % will still develop DGF. 83.8 % of the recipients who will develop DGF are identified. These recipients will have to be precisely monitored after kidney transplantation, making an early identification of graft dysfunction possible. 69.4 % of all positively identified recipients will eventually not develop DGF.

Our study does have limitations. Firstly, our sample size of approximately 500 transplantations is lower than in the existing models. It is known that machine learning techniques generally benefit from a large amount of data, increasing their performance [19]. However, we benefited from the detailed and high-quality peritransplant data that could be collected, which is largely unavailable in registries. Secondly, the incidence of DGF in our cohort is lower than in the existing models. This imbalance is addressed by assigning more weight to the ‘DGF’ class during the learning phase of the predictive models. Thirdly, single-center models limit generalizability. However, we used cross-validation to attenuate the generalization error. Finally, our analysis included most, but not all, of the identified risk factors for DGF.

## Conclusions

Nine distinct types of predictive models for DGF are considered. The discriminative capacities of LDA, linear SVM, radial SVM and LR are the only ones above 80 %. None of the pairwise AUROC comparisons between these models is statistically significant, except linear SVM outperforming LR. Additionally, a method with higher sensitivity is preferred over a method with higher specificity in the prediction of DGF, because the damage of an unnecessary biopsy is outweighed by the potential benefit of an early management. The sensitivity of linear SVM to identify recipients with DGF is amongst the three highest of all models. Due to both reasons, the authors believe that linear SVM is most appropriate to predict DGF.

## Abbreviations

AUROC: Area under the receiver operating characteristic curve
DGF: Delayed graft function
DT: Decision tree
LDA: Linear discriminant analysis
LR: Logistic regression
PPV: Positive predictive value
QDA: Quadratic discriminant analysis
RF: Random forest
SGB: Stochastic gradient boosting
SVM: Support vector machine
